# Dynamical Integration of Language and Behavior in a Recurrent Neural Network for Human–Robot Interaction

**DOI:** 10.3389/fnbot.2016.00005

**Published:** 2016-07-15

**Authors:** Tatsuro Yamada, Shingo Murata, Hiroaki Arie, Tetsuya Ogata

**Affiliations:** ^1^Department of Intermedia Art and Science, Waseda University, Tokyo, Japan; ^2^Department of Modern Mechanical Engineering, Waseda University, Tokyo, Japan

**Keywords:** symbol grounding problem, language learning, human–robot interaction, recurrent neural networks, sequence to sequence learning, dynamical system approach

## Abstract

To work cooperatively with humans by using language, robots must not only acquire a mapping between language and their behavior but also autonomously utilize the mapping in appropriate contexts of interactive tasks online. To this end, we propose a novel learning method linking language to robot behavior by means of a recurrent neural network. In this method, the network learns from correct examples of the imposed task that are given not as explicitly separated sets of language and behavior but as sequential data constructed from the actual temporal flow of the task. By doing this, the internal dynamics of the network models both language–behavior relationships and the temporal patterns of interaction. Here, “internal dynamics” refers to the time development of the system defined on the fixed-dimensional space of the internal states of the context layer. Thus, in the execution phase, by constantly representing where in the interaction context it is as its current state, the network autonomously switches between recognition and generation phases without any explicit signs and utilizes the acquired mapping in appropriate contexts. To evaluate our method, we conducted an experiment in which a robot generates appropriate behavior responding to a human’s linguistic instruction. After learning, the network actually formed the attractor structure representing both language–behavior relationships and the task’s temporal pattern in its internal dynamics. In the dynamics, language–behavior mapping was achieved by the branching structure. Repetition of human’s instruction and robot’s behavioral response was represented as the cyclic structure, and besides, waiting to a subsequent instruction was represented as the fixed-point attractor. Thanks to this structure, the robot was able to interact online with a human concerning the given task by autonomously switching phases.

## 1 Introduction

In recent years, the idea of robots that work flexibly in a human’s living environment has been attracting great attention. An understanding of language is indispensable for them to communicate and work with humans efficiently. In a dynamically changing environment, robots must work autonomously in an online manner while understanding the language shared with humans, in other words, mapping the language to meaning in their situation, such as objects, events, or their intentional behavior. This mapping is not one-to-one but consists of many-to-many relationships characterized by ambiguity and context dependency. This difficulty is known as the “symbol grounding problem” (Harnad, 1990). In order to continue open-ended interaction in the real world, in which events never happen again in exactly the same way, robots must acquire language–meaning relationships by learning from a limited number of experiences and must behave appropriately even in novel situations by generalizing the acquired relationship, as a human does.

To date, especially in the field of developmental robotics (Asada et al., 2009; Cangelosi et al., 2010), there have been some studies that attempted to understand human language and its developmental aspects by constructive means, such as learning robot experiments (Ogata and Okuno, 2013; Hinaut et al., 2014; Zhong et al., 2014). They have investigated integrative learning between language and robot behavior mainly by means of neural network models and have achieved integration with a certain degree of generalization ability in experiments (Sugita and Tani, 2005; Ogata et al., 2007; Tuci et al., 2011; Chuang et al., 2012; Stramandinoli et al., 2012; Heinrich and Wermter, 2014; Yamada et al., 2015). One the other hand, in the field of symbol emergence robotics centered on Japan (Taniguchi et al., 2016), researchers have also dealt with language acquisition and its grounding in meaning for constructive understanding and engineering applications (Roy and Pentland, 2002; Iwahashi, 2003). They mainly build probabilistic models, such as hidden Markov models, latent Dirichlet allocation models, and non-parametric Bayesian models, and train these models in an unsupervised manner, in which the symbol system emerges from exposure to raw data of utterances, motions, and video (Inamura et al., 2004; Iwahashi, 2008; Takano and Nakamura, 2009; Nakamura et al., 2011; Araki et al., 2012). These fields share the notion of “embodied” intelligence, which argues that intelligence, including language use, emerges only from interactions between the internal cognitive system and the external environment mediated by the sensorimotor systems specific to the subject’s body (Pfeifer and Scheier, 1999). Therefore, they often refer to cognitive linguistics perspectives on language, such as usage-based model or thematic role assignment (Sugita and Tani, 2008; Hinaut and Dominey, 2013; Hinaut and Wermter, 2014), about cognitive linguistics, for instance, see Tomasello (2003). The problems discussed by these works include grounding of primitive verbs (nouns) and motions (objects), acquisition of higher level concepts from the primitives, and learning of syntactic structure.

Some models in these researches are able to translate sentences to a corresponding meaning, such as motions, and vice versa, by utilizing an acquired mapping (Ogata et al., 2007; Takano and Nakamura, 2009; Hinaut et al., 2014). Translation algorithm usually consists of phases distinguished in advance as follows: first, a whole sentence is given and recognized, then the translation is conducted, and finally a response is generated. However, in real situations involving collaborative work with other agents, the signs for phase-switching are not always given externally but are, rather, embedded implicitly in the interaction context. Therefore, robots must find these timings autonomously from the interaction context. As one example, consider a simple cooperative interaction task in which a robot is required to respond to a human’s instruction by behaving appropriately. First, the robot receives an instruction. At the end of the instruction, the robot must notice that the instruction has finished, then translate the sentence on a corresponding behavior, move into its own generation phase, and eventually behave appropriately. Moreover, after its own behavior phase, the robot must be able to wait for a subsequent instruction in order to continue the interaction. The robot should acquire the functions for dealing with all of these requirements online in real situations. When the task is changed, different requirements could become important. In brief, for applications to human–robot interaction, robots are required in the learning phase to internally model language–behavior relationships and the temporal patterns of the interaction. Furthermore, during the execution phase, they must retain and process contextual information constantly in order to identify where in the interaction context they are and must utilize the acquired language–behavior relationships in appropriate contexts in an online manner. When learning and grounding methods that satisfy these requirements are established, they will lead to the possibility that we can teach robots to execute collaborative tasks requiring language use, just by giving a certain number of examples of interactions as raw sequential data without any preprocessing to construct explicit sets of language and corresponding behavior. We aim to tackle this problem and propose a novel linking^1^ structure between language and robot behavior that can be used by robots autonomously in appropriate contexts.

To this end, we propose a method that employs a recurrent neural network (RNN), which has recently attracted much interest in the field of natural language processing (NLP) (Mikolov et al., 2010; Bahdanau et al., 2015; Vinyals and Le, 2015; Li et al., 2016). RNNs can extract temporal patterns from sequential data and approximately learn the non-linear function that predicts future states from the input history up to the current state (Elman, 1990). In particular, we take our cue from the method referred to as “sequence to sequence learning” (Sutskever et al., 2014), in which the RNN learns to map a sequence to a corresponding sequence in its forward propagation. Therefore, the trained RNN can deal with tasks, such as translation or a troubleshooting chat, interactively (Vinyals and Le, 2015). To solve the aforementioned problems, we propose an extension of the method so that it trains the RNN to learn both the mapping from a linguistic sequence to a behavioral sequence, and the temporal patterns of the interactive task in its forward propagation. To evaluate our method, we designed an experiment in which a robot must respond to a human’s instructions by behaving appropriately. After training with datasets constructed as a series of temporal flows of human–robot interaction, the robot successfully interacted with a human by autonomously switching recognition, generation, and waiting phases and by utilizing the systematically acquired relationships in appropriate contexts using only forward calculation of the RNN.

This paper is organized as follows. In Section 2, we review the existing studies of learning experiments with the sequence to sequence method and other relevant studies that investigated the characteristic of the dynamical system of RNNs. In Section 3, we propose our method in which the dynamical representations of both language–behavior relationships and human–robot interaction are self-organized on RNN’s dynamical system, and introduce a technique to get a desired representation. We also consider functional hierarchization by introducing a multiple timescale RNN (MTRNN) (Yamashita and Tani, 2008). In Section 4, we explain the task design for the robot experiment that evaluates the effectiveness of our proposed method. In Section 5, we give the experimental results and analyze the internal representations that are formed on the RNN by learning. In Section 6, we compare our method and results with those of other studies, discuss our findings, and present our conclusions.

## 2 Related Works

Many studies have conducted learning experiments related to language (Elman, 1990; Hinoshita et al., 2011; Mikolov et al., 2013) or the integration of language and other modalities, which include not only robot motions but also images (Karpathy and Fei-Fei, 2015; Vinyals and Le, 2015), by means of NN models. Among them, the method that has recently attracted much interest is the method referred to as sequence to sequence learning, in which the RNN model recognizes temporal sequences and generates corresponding sequences in a continuous series of forward calculations without resetting (Sutskever et al., 2014). Here, the forward calculation or forward propagation is the non-linear mapping from input sequences to output sequences optimized by learning. Vinyals and Le (2015) trained an RNN model with long short-term memory (LSTM) units using a large conversational corpus given to the model as sequential data. After learning, the model responded to a human’s questions and had a chat just using its forward calculation. Sutskever et al. (2014) also utilized a similar model for English-to-French translation. Vinyals et al. (2015) combined a convolutional neural network (CNN) and an RNN with LSTM units to generate sentence captions from images in its forward calculation, although their model is not sequence to sequence. This method, which integrates recognition, translation, and generation in successive forward calculations, seems to have an advantage from the perspective of application to practical human–robot interaction because networks trained in this manner can work in an interactive manner by the forward calculation requiring the low calculation cost.

So far, the method of sequence to sequence learning has been hardly imported into robotics. Because of problems, such as phase-switching, that exist in actual human–robot interactions, the method cannot be applied directly. For example, in the study of Vinyals and Le (2015), a human’s turn (question) and a machine’s turn (answer) were separated explicitly by the end of sequence symbol to solve the phase-switching problem. Although this solution would be no problem as long as the application is limited to human–machine conversation, this artificial strategy is not suitable for online human–robot interaction in the real world. Instead, it is desirable that the robot autonomously switches between the phases without any explicit signals. To our knowledge, only Park and Tani (2015) have applied the sequence to sequence method in robotics and conducted experiments in which a robot learned to respond to a human’s imperative gesture with a corresponding gesture in accordance with a semantic rule by means of forward calculation in an RNN. Their model dealt with interaction in a single modality, gesture. In contrast, we deal with the integration of language as a symbolic modality and behavior as a continuous modality. Moreover, in their task, the imperative gestures were mapped into response gestures one-to-one. In contrast, we explicitly deal with environmental changes, which lead to an ambiguous relationship between language and behavior.

Next, to make the explanation of our proposed method in the following section more understandable, we review the workings of RNN in existing works from static and dynamic^2^ perspective. From the static perspective, the internal state of the RNN context layer at a certain time step, namely, the fixed-dimensional vector, whose size is the number of context neural units, is determined by the past input history as well as determining the output sequence after the step. Therefore, in the schema of the sequence to sequence learning, the internal state of the network reaches a certain activation in accordance with the meaning of the received sentence and immediately generated its own response sentence based on the activation. Here, the internal activation at the end of the human’s sentence can be interpreted as a static representation of the meaning of the human’s sentence in the form of an encoding in the fixed-dimensional vector; it also can be interpreted as a static representation of its own following response. In other words, the linking of the human’s sentence to the network’s response is encoded as a static fixed-dimensional vector.

On the other hand, such workings also can be seen from a more dynamic perspective. From the aspect of the network dynamics after learning, the time development on the internal states of the context layer during forward calculation can be seen as a representation of the temporal flow of data. For example, Yamashita and Tani (2008) trained a humanoid robot implemented with an RNN to generate iterative motion patterns. During motion generation, cyclic transitions synchronized with the motions could be seen in the time development of internal states of the context layer. In another experiment by Tani and Ito (2003), multiple attractors, including fixed points and cycles corresponding to various motion primitives, were formed in the internal dynamics of an RNN. As shown in these cases, RNNs can acquire an internal dynamical representation that works synchronously with input/output (I/O) temporal sequences. In particular, the temporal transitions of internal states having attractor structure are robust against noise; thus, it can be applied to practical situations, such as motion generation tasks. Even in the sequence to sequence model, the neural activation continues to dynamically change during the phases of both the human’s sentence and the network response. Thus, by synthesizing the static and dynamic perspectives, we can describe the execution of this communicative task as follows: the input sentence can be linked on the output sentence through a static representation, while the whole of the time development of the internal states synchronously represents the temporal flow of the communication, which consists of the human’s sentence and the subsequent network response.

In the next section, on the basis of the above review of an RNN’s working, we propose our novel linking method in which the dynamics of the RNN represents the interaction pattern and allows the robot to interact with a human by utilizing the acquired relationships in appropriate contexts.

## 3 Materials and Methods

In this section, we describe a novel language–behavior linking structure that can be used in practical interactions by means of an RNN. We also describe some techniques to achieve the linking structure.

### 3.1 Overview of Task and System

**Figure 1:**
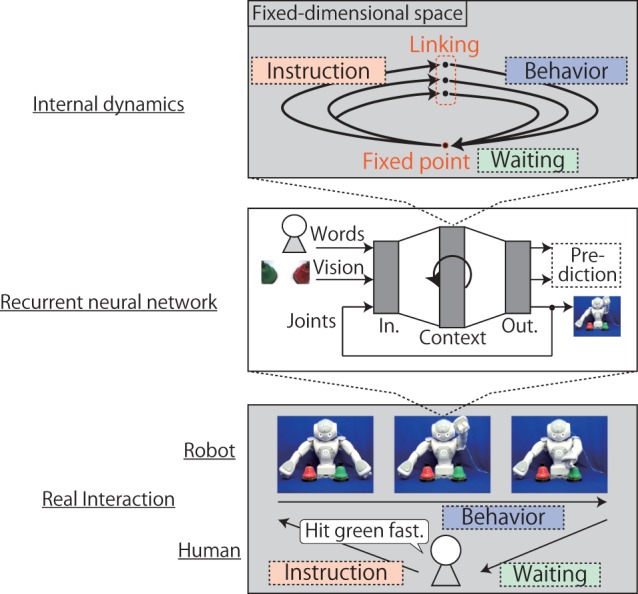
**Representation of interactions by the internal dynamics of the RNN context layer**. First, in the instruction phase, by receiving words input to the network one by one, the internal states of the context layer are activated in the fixed-dimensional space, branching in accordance with the meaning of the words. After the instruction, the internal states reach a certain activation corresponding to the meaning of the sentence. From the activation (i.e., the linking point), the network immediately generates appropriate behavior in subsequent forward calculation, while the internal states move along the second half of the attractor. Eventually, the internal states return to the initial point, which has been formed as a fixed point, and the robot waits for another instruction in a stable state.

In this study, to make the explanation and the evaluation of the method clearer, we consider only one possible situation; a task in which a robot responds to a human’s linguistic instructions by generating appropriate behavior (the bottom panel of Figure 1). In Section 6, however, we explain that our method can be applied to other interactive tasks. In the task, because the mapping from an instruction to the corresponding behavior sometimes requires the robot to use visual information, in other words, because the mapping has a many-to-many relationship, the robot must acquire systematic semantics in order to behave appropriately even in unexperienced situations. Moreover, to interact with a human in an online manner, the robot must also autonomously switch between phases, such as recognition, generation, and waiting, by processing contextual information and use the acquired relationships in appropriate contexts.

For the learning experiment, we use the small humanoid robot NAO made by Aldebaran that has a body corresponding to only the upper half of the human body. The sequential data fed into/out of I/O neural units of the RNN consist of the words, robot vision, and joint angles (the center panel of the Figure 1). Each word is assigned one I/O neural unit; a sentence is represented as a sequence of words. Visual images are got by the robot’s head mount camera. Ten units are also assigned to the robot’s arm joints (ShoulderRoll, ShoulderPitch, ElbowRoll, ElbowYaw, and WristYaw on both the arms). In this setting, the RNN is trained to predict future states of the data. In the evaluation phase after learning, the output of joint angle units is fed not only into the robot as a motor command but also back into the input layer on a subsequent time step. By doing this, we can interpret the sequences generated by output units of joint angles as the robot’s autonomous behavior responding to instructions.

### 3.2 Proposed Method

#### 3.2.1 Dynamical Representation of Interactions by an RNN

In this section, we introduce our essential method, the novel linking structure that allows the robot to interact with a human just by forward calculation in the RNN. On the basis of the review of an RNN’s working in the previous section, we thought that the method of sequence to sequence model could be applied to interactions that require a link between language and robot behavior. We hypothesized that if the link is embedded as a static representation in the middle of the dynamics of an RNN that synchronously represents the temporal flow of interaction, interactions that require the online use of the language–behavior relationships could be achieved. Consider the instruction–behavior task shown in Figure 1. First, in the instruction phase, by receiving the words input to the network one by one, the internal states of the context layer are activated, branching in accordance with the meaning of the words. After the instruction, the internal states reach a certain activation corresponding to the meaning of the sentence. Subsequently, from this activation, which can be interpreted as the linking point, the network immediately generates the appropriate behavior in a subsequent forward calculation. Moreover, the internal states are required to go back to the initial point after the behavior generation in order to receive the next instruction. By acquiring such a cyclic attractor structure that represents recognition and generation, the robot can continue the given interactive task sustainably by autonomously switching between the recognition and generation phases and utilizing the acquired relationship in appropriate contexts using only forward calculation. In this synchronous mechanism, where the interaction context of the robot is continuously represented by the current internal state, the language–behavior relationships are also embedded as a fixed-dimensional vector in the middle of the cyclic attractors. However, in real instruction–behavior tasks, the instructions are not given in a perfectly periodical way. Therefore, the robot has to gain the ability to wait for subsequent instructions after behavior generation. This ability is also acquired by forming a corresponding representation in the RNN’s internal dynamical system. To be more precise, if a fixed-point attractor is formed at the initial point, the robot is able to wait for a human’s instructions in its initial posture.

#### 3.2.2 Training Sequences Constructed as Raw Streams of Interactions

Subsequently, we explain how to construct training sequences to allow the network to acquire the aforementioned internal dynamics that enables the robot to interact. In the scheme of sequence to sequence learning, the temporal structures are learned just by experiencing a certain number of examples in a data-driven manner, by utilizing the back-propagation through time (BPTT) algorithm (Rumelhart et al., 1986). We hypothesized that if there are any contextual patterns of interaction, such as a series that consists of instruction, behavior, and waiting in this order, in target data, the model would acquire the temporal patterns as well as the linking relationships. The details are as follows. First, the training sequences must be constructed as successions of instruction and corresponding behavior for the network to self-organize^3^ a representation as a continuous time development of the internal states in forward calculation, as in sequence to sequence learning. Furthermore, because the current task requires the robot to repeat the interaction, the training sequences must concatenate a number of interaction episodes. Constructing the target in such a way, the cyclic attractors that enable the robot to respond to a human’s instruction not only once but any number of times can be formed. Finally, the interval length of episodes, namely the number of time steps from the robot behavior to the subsequent instruction, must be variable for the network to form a fixed-point attractor that allows the robot to wait stably for instructions. In brief, trained by target sequences that are constructed to include various aspects of the given task without abstracting them, the network can acquire the internal dynamical representations that give it the ability to deal with those temporal aspects.

### 3.3 Employed Neural Network Model: MTRNN

#### 3.3.1 Hierarchical Functionalization in an MTRNN

In this section, we introduce the learning model employed. In the current case, although the task described at the episode level is just a simple repetition of instruction, behavior, and waiting phases, the raw data level is more complicated: first the network gains the appropriate activation by receiving word inputs and visual information and integrating them while remaining at rest in the waiting posture; after that, the network immediately generates the detailed joint angle sequence of various motions. Therefore, to cope with such a task, the RNN has to learn not only representing patterns at the episode level with long timescale dependencies (i.e., the global context of interactions as cyclic attractors and the link of language to behavior as a branching structure) but also translating the current complicated I/O flows at the raw data level into the aforementioned internal representations and vice versa. Assuming that the current interaction context is constantly represented by the current internal states, this transformation itself is a function with short timescale dependencies.

**Figure 2:**
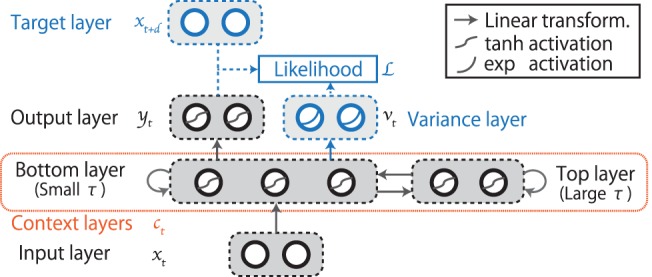
**The structure of the MTRNN with two stacked context layers**. The data flow fed into/out of the I/O layer of the RNN consists of the words, robot vision, and joint angles. Blue-colored parts are used only for learning and explained in detail in Section 3.3.2.

To deal with both timescales, we employ an MTRNN that has multiple context layers working with different time constants (Figure 2). The MTRNN can hierarchically self-organize functions working at different time scales on different layers (Hinoshita et al., 2011). For example, Yamashita and Tani (2008) conducted a robot experiment in which a robot equipped with an MTRNN was trained to learn motion sequences that consisted of various motion primitives. Implemented using two context layers, one that had a small time constant and one that had a large time constant, the network hierarchically self-organized its internal dynamics, working synchronously with the primitives in the former and representing the orders of primitives in the latter. In the current case, the MTRNN with two stacked context layers allows the robot to deal with both of the current I/O flows and the global context. Specifically, the cyclic representations that correspond to the global context of the interaction and that embed language–behavior link are self-organized in the top layer that has a large time constant, and the representations more directly corresponding to the detail of the current I/O flows are self-organized in the bottom layer that has a small time constant. In other words, the bottom layer facilitates the bidirectional non-linear transformation between I/O flows and the dynamical representations of the interaction in the top layer. During the instruction phase, the bottom layer receives the word input and the visual information. It then propagates the information to the top layer, so that the top layer can be activated along the correct attractor corresponding the meaning. This is the bottom-up working. In contrast, during the behavior phase, the transitions along the second halves of the attractors in the top layer dynamics can be transformed into various temporal sequences of joint angles in the output layer through the bottom layer. This is the top-down working. By working in such a hierarchically functionalized manner, the network allows the robot to interact with a human using forward dynamics.

#### 3.3.2 MTRNN Formulation

In this section, we explain the mathematics of the MTRNN employed. Usually, squared errors are used as the loss function for the learning of normal MTRNNs. However, in cases that use squared errors as the loss function, when the target sequence has noisy or unpredictable parts, learning might collapse due that the network attempts to forcibly learn these parts. To avoid this problem, this study employs an extended MTRNN introduced by Murata et al. (2015). This model learns to predict not only the external states at a future time step but also their uncertainty as variance. Thanks to the likelihood function defined as presuming the uncertainty in target sequences, the errors back-propagated to the learnable parameters can be decreased with respect to such unpredictable parts by optimally predicting the uncertainty. Therefore, this model can stably learn the structure of training data.

In the forward calculation, the internal state of the *i*th neural unit on each of bottom, top, output, variance layer at time step *t* (*u_t,i_*) is computed by the following equations:

(1) $$u_{t,i}=\left\{\begin{array}{l} \left(\text{1}-\frac{1}{\tau_{\text{B}}}\right)u_{t-1,i}+\frac{\text{1}}{\tau_{\text{B}}}\left(\sum_{j\in I_{\text{I}}}w_{\mathrm{ij}}x_{t,j}+\sum_{j\in I_{\text{B}}}w_{\mathrm{ij}}c_{t-1,j}+\sum_{j\in I_{\text{T}}}w_{\mathrm{ij}}c_{t-1,j}+b_{i}\right)\;\left(1\leq t\wedge i\in I_{\text{B}}\right),\\ \left(1-\frac{\text{1}}{\tau_{\text{T}}}\right)u_{t-1,i}+\frac{\text{1}}{\tau_{\text{T}}}\left(\sum_{j\in I_{\text{B}}}w_{\mathrm{ij}}c_{t-1,j}+\sum_{j\in I_{\text{T}}}w_{\mathrm{ij}}c_{t-1,j}+b_{i}\right)\;\left(1\leq t\wedge i\in I_{\text{T}}\right),\\ \sum_{j\in I_{\text{B}}}w_{\mathrm{ij}}c_{t,j}+b_{i}\;\left(1\leq t\wedge i\in I_{\text{O}}\right),\\ \sum_{j\in I_{\text{B}}}w_{\mathrm{ij}}c_{t,j}+b_{i}\;\left(1\leq t\wedge i\in I_{\text{V}}\right), \end{array}\right.$$

where *I*_I_, *I*_B_, *I*_T_, *I*_O_, and *I*_V_ are the neural unit index sets of the input, bottom, top, output, and variance layers, respectively; *τ*_B_ and *τ*_T_ are the time constants for the bottom and top layers, respectively; *w_ij_* is the connection weight from the *j*th presynaptic unit to *i*th postsynaptic unit; *b_i_* is the bias of the *i*th unit; *x_t,j_* is the *j*th element of the input vector at time step *t*. The internal states of respective layers are activated non-linearly as follows:

(2) $$c_{t,i}=\tanh\left(u_{t,i}\right)\;\left(0\leq t\wedge i\in I_{\text{B}}\right),$$

(3) $$c_{t,i}=\tanh\left(u_{t,i}\right)\;\left(0\leq t\wedge i\in I_{\text{T}}\right),$$

(4) $$y_{t,i}=\tanh\left(u_{t,i}\right)\;\left(1\leq t\wedge i\in I_{\text{O}}\right),$$

(5) $$v_{t,i}=\exp\left(u_{t,i}\right)\;\left(1\leq t\wedge i\in I_{\text{V}}\right).$$

As defined by equation (1), the top layer and I/O layer are not connected directly, but input signals can be conveyed to the top layer through the bottom layer, and the activation of the top layer also controls the output through the connections in the opposite direction. The target data for learning are defined by:

(6) $${ŷ}_{t,i}=x_{t+d,i}.$$

The prediction constant *d* is the fixed parameter that determines what time step away to be predicted. This parameter is adjusted to the sampling rate of the recorded sequential data and used in both learning and evaluation phase. If *d* is set to a small value, the prediction error to be back-propagated tends to be influenced by noise. In contrast, set to a large value, the network can not respond to input signals with required promptness. The network is trained by maximizing the following likelihood function *L*:

(7) $$L=\prod_{t=1}^{T}\prod_{i\in I_{\text{O}}}\frac{1}{\sqrt{2\pi v_{t,i}}}\exp\left(-\frac{{\left({ŷ}_{t,i}-y_{t,i}\right)}^{2}}{2v_{t,i}}\right),$$

where *T* is the length of the sequence. This formulation means that this model presumes that target sequences are generated by adding time-varying Gaussian noises to the source sequences and learns to predict the mean (source) and the variance (noise) in each time step. In the learning, the log likelihood function ln *L* is back-propagated to all the past internal states without truncation to train all the learnable parameters ***θ*** by utilizing the BPTT algorithm. The parameters are updated by the gradient ascent method as follows:

(8) θ(n)=θ(n−1)+αΔθ(n),

(9) $$\Delta \theta \left(n\right)=\frac{\partial \ln L}{\partial \theta }+\eta \Delta \theta \left(n-1\right),$$

where *n* is the learning step, *α* is the learning rate, and *η* is the momentum term. Please refer to Murata et al. (2015) for details of the gradient calculation. Note that the variance layer is used only in the training phase for stable learning and then ignored in the evaluation phase. The source code of this learning model is available at https://github.com/ogata-lab/SCTRNN.

## 4 Experimental Design

To evaluate whether our linking method enables a robot to interact with a human and whether the expected hierarchical structure can be self-organized, we conducted a robot experiment, in which a robot was trained to respond to a human’s linguistic instructions by generating appropriate behavior using visual information if necessary.

### 4.1 Task Design

Here, we specify the task imposed on the robot. The interactive task assigned to the robot is as follows. First, two bells colored red, green, or blue are placed in front of the robot to the left and right. Then, a human instructs the robot by a three-word sentence that consists of (P) Verb + Position + Adverb (e.g., “Point left slowly”) or (C) Verb + Color + Adverb (e.g., “Hit red fast”), where the objective words and adverb indicate one of the bells and the motion speed, respectively. When the two bells have the same color, the robot cannot determine which bell is indicated by an instruction of the pattern (C), so a four-word sentence that consists of (C′) Verb + Color + Position + Adverb (e.g., “Point blue right fast”) is used in these cases. Receiving the sentence, the robot immediately starts to generate behavior corresponding to the instruction. After the behavior generation, the robot waits for a subsequent instruction. We call this chunk of interaction an “episode.” In this task setting, the number of possible episode patterns is 144, that is, the combination of 8 behaviors (POINT, HIT) × (LEFT, RIGHT) × (SLOWLY, FAST), 9 bell arrangements (R,G,B) × (R,G,B), and 2 instructions (P, C or C′).

### 4.2 Target Data

**Figure 3:**
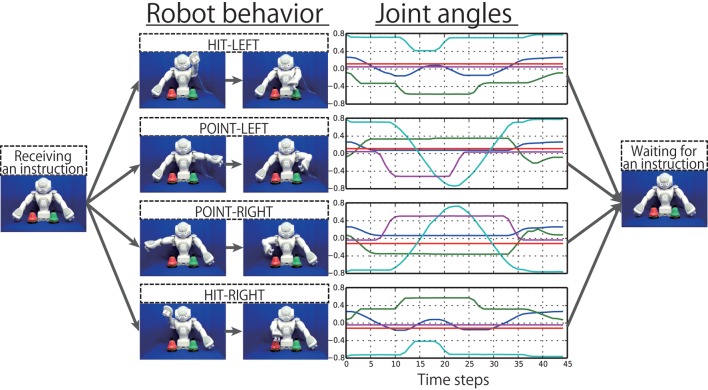
**The behaviors to be generated by the robot responding to the human’s instructions**. Each behavior is generated either “SLOWLY” or “FAST,” which take approximately 45 and 30 time steps, respectively. For each behavior, only the five joint angles on the moving arm are plotted.

**Figure 4:**
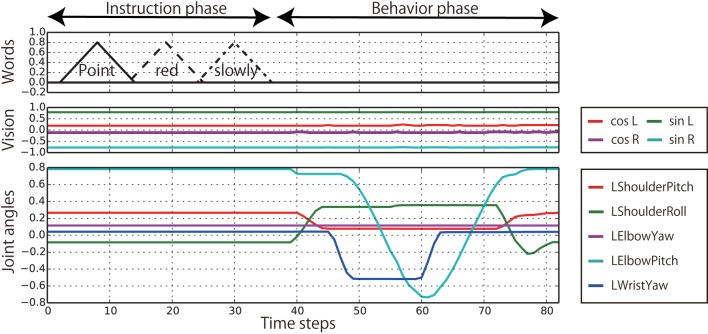
**An example of a target sequence representing an episode**. In this episode, the robot generates the “POINT-LEFT-SLOWLY” behavior after receiving the instruction “Point red slowly” when the bell arrangement is Red–Green from left to right. Only the five joints of the left arm are plotted.

The target sequences were collected as follows. First, the behavior sequences were obtained by actually running the robot (Figure 3). Each sequence was recorded as a sequence of 10-dimensional joint angle vectors by executing the programs controlling the robot arms along predefined trajectories. The sequences were recorded every 240 ms. The slow behaviors and the fast behaviors took approximately 45 and 30 time steps, respectively. The recorded angles were normalized so that the movable ranges were from −0.8 to 0.8. The visual images were simultaneously recorded by the robot’s built-in camera and converted into 4-dimensional vectors consisting of sine and cosine of hues of bell colors, normalized by multiplying them by 0.8. After recording all the combinatorial sequences of behaviors and bell colors, the instructions were prefixed to them on a computer. The instructions are represented as sequences of 9-dimensional vectors, each element of which corresponds to one word (Point, Hit, Left, Right, Red, Green, Blue, Slowly, and Fast). The instructive sentences consist of a series of words that are expressed by triangle activations that reach the top, 0.8, in six steps, and go back down to zero in six steps. Here, the joint angles and visual information in the instruction phases were set to the same values as the initial values of the following behavior phase. Therefore, the robot stays in the initial posture during the instruction phases, and the bell colors are not changed in an episode, although some noise and fluctuation that have been added in data recording can be included. In this way, all of the 144 episode patterns were created as sequences of 23-dimensional vectors (Figure 4), available at http://ogata-lab.jp/projects/cognitive-robotics-group.html.

Subsequently, we made long sequences concatenating a number of the episodes in random order for the RNN to achieve the ability to sustainably continue the interaction by forming a cyclic structure. The intervals between episodes varied from 3 to 25 steps to form a fixed-point attractor enabling the robot to wait for subsequent instructions. Note that no explicit phase-switching signs are included in the target sequences. The network extracts the implicit interaction pattern from the experiences and acquires the ability to autonomously switch phases by learning. We made three training datasets. Dataset 1 comprised 72 sequences, each of which concatenates 20 episodes; all the possible episodes were included at least once (144/144). Dataset 2 also comprised 72 sequences concatenating 20 episodes. However, half of the possible patterns were excluded from the set for the generalization test (Table 1, 72/144). Dataset 3, similarly, comprised 72 sequences concatenating 20 episodes; only one-third patterns were included in the set (Table 2, 48/144). We executed learning and evaluated the results independently for each set.

**Table 1:** **Episodes included in dataset 2 (72/144)**.

Behavior	Bell colors								
	R–R	R–G	R–B	G–R	G–G	G–B	B–R	B–G	B–B
POINT-L-SLOWLY	P	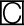	P	C	P	C	P	C	P
POINT-L-FAST	C′	P	C	P	C′	P	C	P	C′
POINT-R-SLOWLY	C′	P	C	P	C′	P	C	P	C′
POINT-R-FAST	P	C	P	C	P	C	P	C	P
HIT-L-SLOWLY	C′	P	C	P	C′	P	C	P	C′
HIT-L-FAST	P	C	P	C	P	C	P	C	P
HIT-R-SLOWLY	P	C	P	C	P	C	P	C	P
HIT-R-FAST	C′	P	C	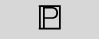	C′	P	C	P	C′

*Two possible instructions that instruct the robot to generate a certain behavior (row) in a certain bell color arrangement (column) exist: that is, (P) position word instruction and (C) or (C′) color word instruction. In dataset 2, the robot experiences one of them for each combination of bell arrangement and behavior. For example, when the bell arrangement is Red–Green, the robot experiences only the (C) color word instruction to generate the POINT-LEFT-SLOWLY behavior (1st row, 2nd col); when the bell arrangement is Blue–Red, the robot experiences only the (P) position word instruction to generate the HIT-RIGHT-FAST behavior (8th row, 4th col)*.

**Table 2:** **Episodes included in dataset 3 (48/144)**.

Behavior	Bell colors								
	R–R	R–G	R–B	G–R	G–G	G–B	B–R	B–G	B–B
POINT-L-SLOWLY		C	P		P	C	P	C	
POINT-L-FAST	C′	P	C		C′			P	C′
POINT-R-SLOWLY	C′			P	C′	P	C		C′
POINT-R-FAST	P	C		C			P	C	P
HIT-L-SLOWLY	C′		C	P			C	P	C′
HIT-L-FAST	P			C	P	C		C	P
HIT-R-SLOWLY		C	P	C		C	P		P
HIT-R-FAST		P	C		C′	P	C	P	

*The RNN did not experience any instructions in black-painted situations*.

### 4.3 Performance Evaluation Method

We created another dataset for evaluation. The evaluation dataset includes all the possible episode patterns. In the evaluation, only the instructions and visual information are input to the trained network externally. In contrast, the input units of joint angles receive values generated by the corresponding output units *d* steps before. By calculating forward in such a manner, we can interpret the sequences generated by output units corresponding to joint angles as the robot’s autonomous behavior. Here, the evaluation is conducted by simulation on a computer, just conducting the forward calculation with the dataset, without the real robot. The performance is evaluated by comparing the generated values of joint angles with the correct values, using the root-mean-square error (RMSE) per joint per time step. In the evaluation, the order of episodes is changed from the training datasets. If the network acquired the temporal patterns as a systematic mapping from the instructions to corresponding behaviors rather than by rote memorization of whole sequences that concatenate a number of episodes, the network would be able to behave appropriately in situations with differently ordered episodes. We also evaluate the waiting ability for instructions by using the other dataset in which the intervals between each episode are set to 100 steps. The performance is also evaluated by using the RMSE between the joint angles generated during waiting phases and those of the initial posture.

## 5 Results

**Figure 5:**
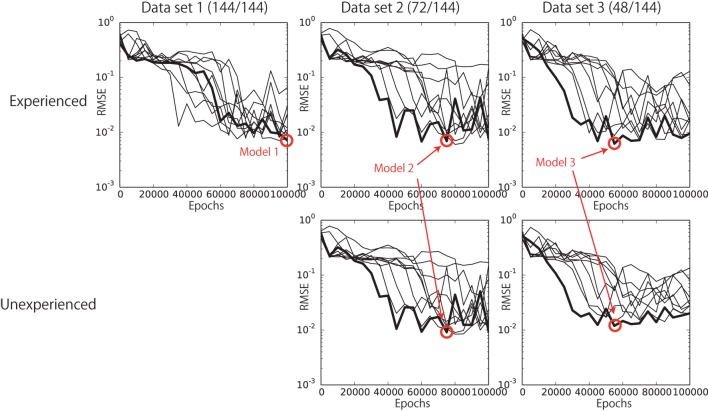
**The learning progress with respect to each dataset**. Each line corresponds to a learning process determined by one random seed. The red circles indicate the networks that scored the best performance among all the used random seeds and learning epochs. We call them Model 1, Model 2, and Model 3.

The network setting employed in the current experiment is as follows. The numbers of neural units in the bottom and top layers were *N_B_* = 80 and *N_T_* = 30, respectively. The time constants were *τ_B_* = 2 and *τ_T_* = 12, respectively. The prediction constant *d*, the momentum term *η*, and the number of training iterations were set to 4, 0.9, and 100,000, respectively. The learning rate *α* was set to 0.1 at the beginning of the learning and adaptively updated during learning process by using the algorithm introduced by Namikawa and Tani (2010). In hyper-parameter search phase, we tried a number of hyper-parameter combinations of *N_B_* = {60, 80}, *N_T_* = {20, 30}, *τ_B_* = {2, 3, 6}, *τ_T_* = {12, 15, 20}, and *d* = {2, 4}. These parameter candidates for trial were empirically determined based on some previous studies that employed NAO and RNN (Murata et al., 2015; Yamada et al., 2015). From these candidates, we carried out the parameter search by try-and-error and eventually chose the above parameter set, which scored the best results among the tried sets. The training was conducted 10 times from randomly initialized learnable parameters with respect to each of the training datasets independently. We evaluated the performance of the trained networks every 5000 epochs. Figure 5 shows the learning processes with respect to each dataset. Each line corresponds to one random seed. Although the learning progressed unstably, the tendency of RMSE decrease relating to both experienced patterns and unexperienced patterns can be shown. In this section, we show the results of the networks that scored the best performance among all the used random seeds and learning epochs (red circles in Figure 5). We call the best network trained by dataset N “Model N.”

### 5.1 Performance of Tasks

#### 5.1.1 Behavior Generation

**Figure 6:**
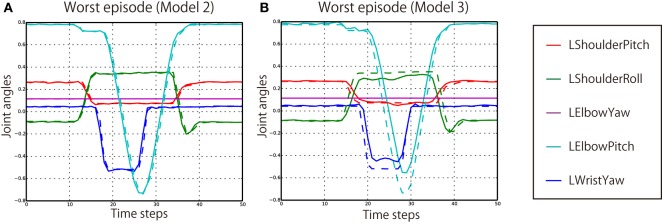
**(A)** The worst unexperienced episode that scored the largest RMSE (0.0203) in the evaluation of Model 2. The solid lines are generated joint angles, and the broken lines indicate correct angles. **(B)** The worst unexperienced episode that scored the largest RMSE (0.0543) in the evaluation of Model 3. Only the five joints on the left arm are plotted, because the robot generated POINT-LEFT-FAST behavior in these episodes.

The quantitative results of the behavior generation performance are shown in Table 3. All the models succeeded in behaving appropriately in all the experienced episode patterns. The overall RMSEs per joint per time step during behavior generation were 0.00706, 0.00691, and 0.00624, respectively. Even in the worst episode, the RMSE was only 0.01173. Here, in the cases of Models 2 and 3, in which a number of episode patterns were not experienced in learning phase, the network seems to more fit to the experienced patterns. The RMSEs of unexperienced situations were a little worse than those of experienced situations. However, by comparison of the generated joint angles and correct ones, it was confirmed that the generated behavior was rather similar to the correct behavior even in the worst episode, as shown in Figure 6.

**Table 3:** **Performance of behavior generation**.

	Experienced (train)			Unexperienced (test)		
	All	Worst	SD	All	Worst	SD
Model 1 (144/144)	0.00706	0.01173	0.00176	–	–	–
Model 2 (72/144)	0.00691	0.00983	0.00150	0.00908	0.02032	0.00308
Model 3 (48/144)	0.00624	0.00790	0.00084	0.01193	0.05433	0.00703

*“All” indicates the overall RMSE per joint per time step during evaluation. “Worst” indicates the RMSE in the worst episode during evaluation. “SD” is of the RMSE of each episode during evaluation. Note that these performances were achieved by the best networks trained from datasets 1–3, corresponding to red circles in Figure 5*.

#### 5.1.2 Waiting Ability

Next, we evaluated the ability to wait for instructions. The results showed that after every behavior generation, the joint angles returned to the initial posture and kept the posture until a subsequent instruction was input. The RMSEs during the waiting phase were 0.00581, 0.00376, and 0.00324 for Model 1, Model 2, and Model 3, respectively. It was also confirmed that after waiting for a long time, the robot could respond to an instruction by generating an appropriate behavior. Thus, the robot had acquired the ability to wait for instructions.

As these results, the robot could continue to interact online with a human with regard to the given task by utilizing acquired relationships in appropriate contexts.

### 5.2 Analysis of Internal Dynamics

#### 5.2.1 Comparison between the Top Dynamics and the Bottom Dynamics

**Figure 7:**
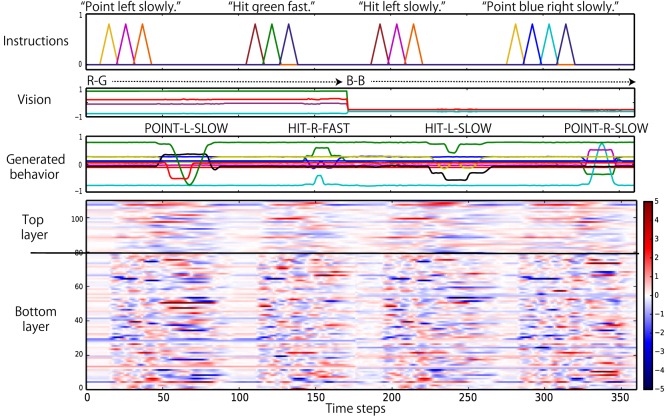
**Comparison of the internal dynamics of the bottom and top layers during evaluation**. The *y*-axis of the color map is the index of the neural units. When the internal state of a unit at a particular time step is more (less) than +5.0 (−5.0), the edge color is provided. The internal states of the bottom layer change more quickly, in general, than the internal states of the top layer.

In previous subsection, we confirmed that the trained network was autonomously able to behave appropriately in the current task. Next, we conducted analyses of the network dynamics and its representations in each context layer. First, we roughly compared the dynamical changes of the internal states of the bottom layer with the top layer. Figure 7 shows that, as expected, the internal states of the bottom layer change more quickly than those of the top layer.

#### 5.2.2 Details of the Top Layer Dynamics

**Figure 8:**
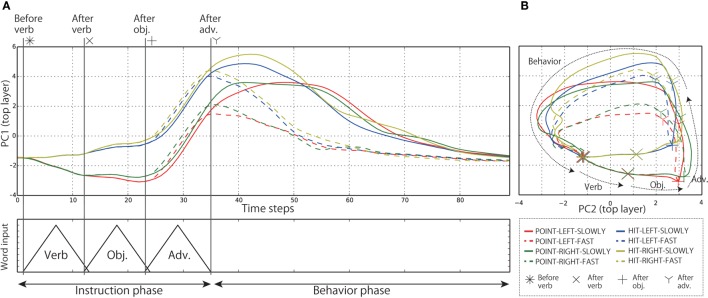
**(A)** The time development of the internal states in the top layer in the PC1 direction. Each line expresses the time development averaged over all the episodes in which each behavior was generated during evaluation. The episodes in which the instruction consists of four words were excluded. The internal states of the top layer develop along branches corresponding to the meaning of the input words. **(B)** The same time development projected onto PC1–PC2 space. The cyclic structure directly representing the temporal flows of interaction, which consist of the repetition of instruction recognition and behavior generation, can be seen. This representation corresponds to the top panel of Figure 1.

**Figure 9:**
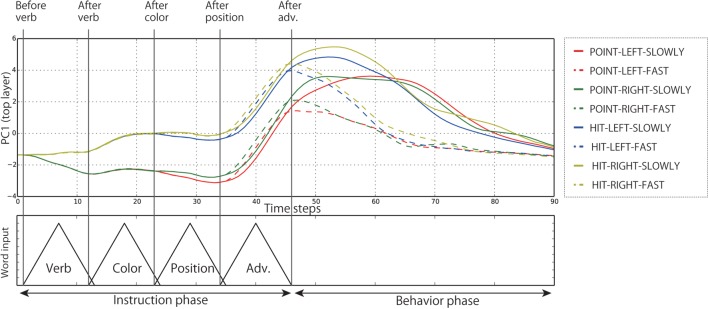
**The time development of the internal states in the top layer in the PC1 direction**. Each line expresses the time development averaged over all the episodes in which each behavior was generated during evaluation. Note that only the episodes in which the instruction consists of four words were included. In the cases of 4-word instruction, the robot cannot identify which of the bells the color word indicates. In these cases, the internal states first branched by receiving the verb but were not bifurcated by the following color word. After that, they were bifurcated twice by a position word and an adverb, and eventually reached the appropriate activation.

Subsequently, in order to analyze the internal representations of both the context layers in detail, we visualized the internal states during the evaluation by projecting them from the high-dimensional space to visualizable subspaces by means of principal component analysis (PCA). Below, we show the results of the analysis of Model 1. The left top panel of Figure 8 shows the time development of the internal states of the top layer during the interaction episodes in the PC1 direction; the contribution ratio (CR) is 28.3%. Each line indicates the average time development for all the episodes in which each of the eight behaviors was generated during evaluation. However, the episodes in which the instruction consists of four words were excluded. By receiving the instruction, the internal states of the top layer branch in accordance with the words. During instruction phase, the internal states developing along different branches for different words (e.g., point or hit) maintain these different transitions without merging together. In other words, the input history of words can be retained as internal states of the top layer. After bifurcating three times (POINT or HIT, LEFT or RIGHT, SLOWLY or FAST), the internal states reach eight different activations corresponding to the respective behaviors. From the points that represent links from instructions to behaviors, the network immediately moves into the behavior phase. In addition, the right top panel of Figure 8 shows the same time development projected onto the PC1–PC2 space, instead of folding up the time axis. By visualizing in this way, it is clearly seen that the cyclic attractors that directly represent the temporal flows of instruction–behavior episodes as the cycles were acquired in the forward dynamics. After behavior phase, the internal states reach the initial point again (asterisk). Thanks to this cyclic dynamics, the robot could continue to interact with the human in the current task. The autonomous phase-switching from recognition to generation can be achieved in a series of forward calculations without any explicit cues. Incidentally, in the cases of 4-word instruction (e.g., “Point red left slowly”), the robot cannot identify which of the bells the color word indicates. In these cases, the internal states first branched by receiving the verb but were not bifurcated by the following color word. After that, they were bifurcated twice by a position word and an adverb, and eventually reached the appropriate activation and immediately generated the corresponding behavior (Figure 9).

**Figure 10:**
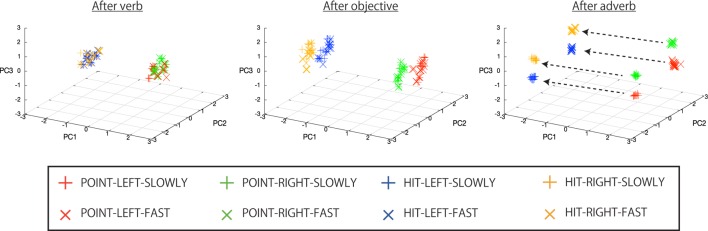
**The internal states after verb, after objective, and after adverb input, from left to right, respectively, projected onto PC1–PC3 space (CR is 52.2, 40.0, 8.5%, respectively)**. The cluster structure topologically organized in accordance with the input words can be seen. The broken lines in the right graph roughly indicate the vectors that connect average linking points of two behaviors differing in only the verb element (e.g., HIT-LEFT-SLOWLY and POINT-LEFT-SLOWLY), that is, the POINT-HIT axis. The minimum cosine between two of the four vectors calculated in the original 30-dimensional space was 0.972, indicating that they are almost parallel to each other. In the cases of the LEFT-RIGHT axis and the SLOWLY-FAST axis, the minimum cosines are 0.938 and 0.977, respectively. In contrast, the cosines (1) between the POINT-HIT axis and the LEFT-RIGHT axis, (2) between the POINT-HIT axis and the SLOWLY-FAST axis, and (3) between the LEFT-RIGHT axis and the SLOWLY-FAST axis were 0.079, 0.075, and 0.035, respectively, indicating that these axes are close to orthogonal.

As one of the important things, when the instruction was the type (C), the course that the internal states should develop along differs according to the arrangement of the bells. For example, the word of “red” can mean either left or right depending on which side the red bell is. Even in such cases, the network was able to choose the correct branch by learning the relationship between the bell colors and color words. Note that because the branching structure is realized by a dynamical system, the trajectory of the internal states is not perfectly identical in each episode, having some variance from the average trajectory. This is also caused by the influence of the previous episode and the perturbation by the visual fluctuation. Figure 10 shows this fact. The graphs in Figure 10 show the internal states after verb, after objective, and after adverb input, projected onto the PC1–PC3 subspace (CR is 52.2, 40.0, 8.5%, respectively, and PCs were extracted by using only the internal states on the top layer just after the instruction phase). The plot types differ in accordance with the behavior to be generated in the episode. The doubling of clusters by receiving words can be seen. Eventually, after the adverb, the eight clusters that correspond to the eight behaviors appear. Thus, the link from instructions to behaviors is actually achieved as a cluster structure.

Here, these clusters have a systematic structure in the topology. To be more exact, the clusters are arranged on the vertices of the parallelepiped, whose axes correspond to POINT-HIT, LEFT-RIGHT, SLOWLY-FAST, respectively. For example, the four broken lines in the right-hand graph of Figure 10 roughly indicate the vectors that connect average link points of two behaviors differing in only the verb element (e.g., HIT-LEFT-SLOWLY and POINT-LEFT-SLOWLY). The minimum cosine between two out of four vectors calculated in the original 30-dimensional space was 0.972, indicating that they are almost parallel. In the cases of the LEFT-RIGHT axis and the SLOWLY-FAST axis, the minimum cosines are 0.938 and 0.977, respectively. In contrast, the cosines (1) between the POINT-HIT axis and the LEFT-RIGHT axis, (2) between the POINT-HIT axis and the SLOWLY-FAST axis, and (3) between the LEFT-RIGHT axis and the SLOWLY-FAST axis were 0.079, 0.075, and 0.035, respectively, indicating that these axes are almost orthogonal. This parallelepiped was developed from the 9-word inputs that were orthogonal to each other through a series of branching dynamics.

**Figure 11:**
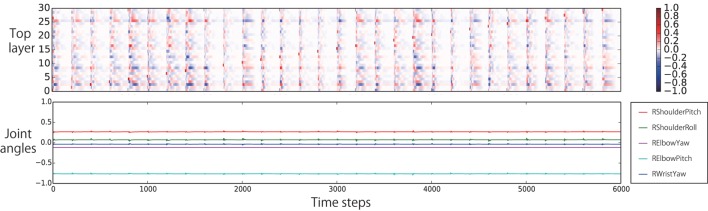
**After behavior generation, instead of giving an subsequent instruction, we added a small perturbation (+1.0) to every neural unit in the top layer every 200 time steps (indicated by orange dots)**. The top panel shows the difference of the internal states computed with the values of the fixed point. White-colored cells correspond to the values of the fixed point. Though the perturbations spread throughout the layer once, the internal states converged again. As shown in the bottom panel, the output joint angles that keep the waiting posture were little influenced by the perturbations.

Last, it was confirmed that the robot can wait stably for instructions in its initial posture, thanks to a stable characteristic of the initial point. This characteristic was evaluated as follows. After behavior generation, the forward calculation was continued with the noiseless input of visual information and the autonomous looped input of joint angles without any instruction inputs. After a large enough number of time steps, the internal states of the top layer converged to a fixed-point attractor. Average reduction rate per time step was approximately 97–98%. After convergence, we added a small perturbation to every neural unit in the top layer one by one (Figure 11). Though the perturbations spread throughout the layer once, the internal states converged again. Thanks to such stable dynamics self-organized on the initial point, the ability to wait stably for instructions in its initial posture was achieved.

Taken together, in the top layer, the dynamical structure working synchronously with the temporal flows of interaction was self-organized. The recognition, generation, and waiting phases were implicitly represented as parts of the attractors. The link from language to behavior was embedded as a topologically organized cluster structure that could be reached by time development along branches corresponding to the words.

#### 5.2.3 Details of the Bottom Layer Dynamics

**Figure 12:**
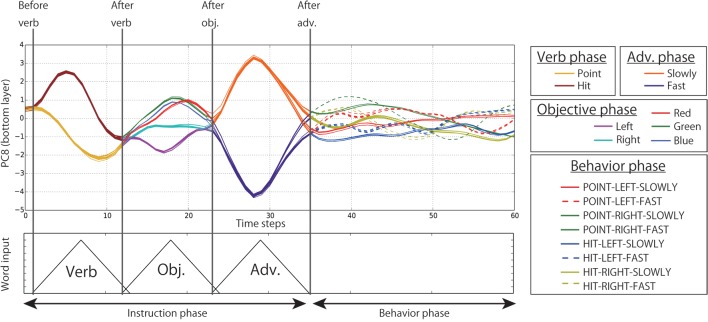
**The time development of the internal states in the bottom layer in the PC8 direction (CR is 3.0%)**. Each line indicates the time development during one episode. The lines are colored differently in verb, objective, adverb, and behavior phases. This figure shows that the internal states of the bottom layer are activated in accordance with the current I/O flows, and past information quickly vanishes from the layer.

We also visualized the dynamics of the internal states of the bottom layer by means of PCA. Figure 12 shows that the neural units of the bottom layer do not retain memory for a long duration, rather they take states corresponding to the current I/O values. First, during the verb phase, the time development of the internal states on the bottom layer differs depending on the verb (point or hit). After moving into the objective phase, the information about the verb input vanishes. Instead, the internal states are activated corresponding to the current input objective (left, right, red, green, or blue). The phase shift from objective to adverb is similar. Instead of vanishing from the bottom layer, the information is fed to the top layer and is retained as mentioned above. In contrast, during the behavior phase, the information retained in the top layer is fed down to the bottom layer. By receiving information flows from the top layer, the internal states of the bottom layer go along different trajectories in accordance with the behavior to be generated. Incidentally, the reason that we plot the time development in the direction of PC8 is that, in this component, the information about verbs, objectives, adverbs, joint angles was included rather evenly, so these facts can be seen easily. Although the higher CR components also showed the similar tendency, they concurrently tended to mainly represent a specific modality. For example, we confirmed that PC1–4 mainly represented the joint angles.

**Figure 13:**
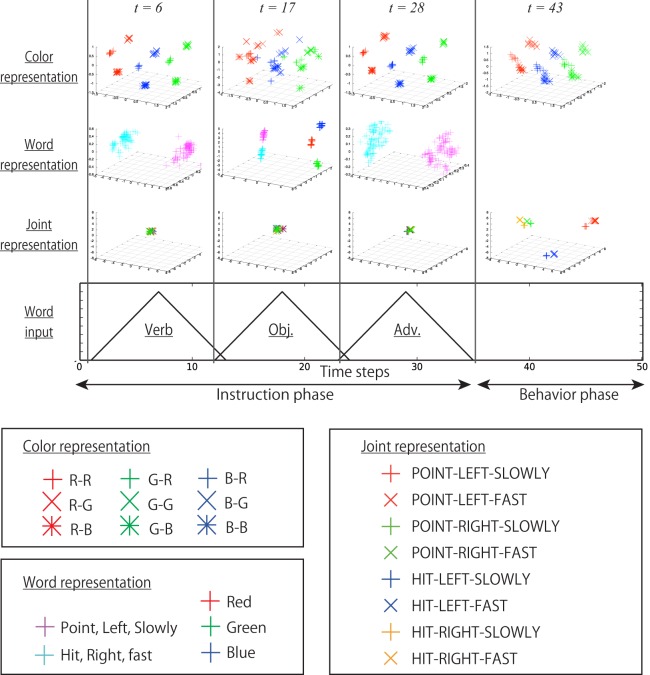
**The internal states of the bottom layer at various time steps during interaction episodes projected onto various subspaces: in other words, the static representations**. The times *t* = 6, 17, 28 correspond to the tops of the triangle input of words. The time *t* = 43 corresponds to the 10th time step after the instruction phase. In the first row, the information about the bell colors is constantly reflected. Note that in the objective phase, the cluster structure is deformed because the integration of bell color and a color word is required. In the space shown in the second row, the cluster structure corresponding to the current word input is shown. In the space of the third row, the information about joint angles is mainly represented.

We analyzed the bottom layer in more detail. Figure 13 shows the internal states at various time steps during interaction episodes projected onto various PC subspaces. In other words, they are static internal representations at various times in the interaction context. The analysis showed that the multimodal information of the current I/O was topologically embedded separately in these spaces. In the space shown in the first row, the information about bell colors is constantly reflected. In the spaces on the second row, the cluster structure corresponding to the current word input is shown. Note that these three graphs on the second row are different subspaces. The graphs on the third row show the internal states projected onto the space mainly representing joint angles. During the instruction phase, the robot keeps its initial posture; thus, the internal states are not activated in this space, and they stay in the vicinity of the initial point. After switching into the behavior phase, the internal states are activated in this space in accordance with the behavior to be generated.

Synthesizing these results with the analysis of the top layer, the working of the whole of the network is as follows. The global representations involved in the whole context of interaction were self-organized in the top layer with slow dynamics. The linking structure was simultaneously embedded in the middle of the dynamics. However, the details of the I/O streams, including multimodal information, are more complicated than the representations visualized by the analysis of the top layer. Thus, the bottom layer, which can change its internal states more drastically, translates between the I/O temporal flows and the top layer representations. In the instruction phase, the bottom layer receives the words and visual information and feeds them into the top layer so that the top layer reaches an appropriate activation. This is the bottom-up working. In the behavior phase, the network works in the top-down manner. Transitions along the second halves of the attractors are transformed into detailed flows of joint angles by a high-dimensional non-linear transformation through the bottom layer. Because the functions that work on different timescales were hierarchically self-organized on their respective layers, the whole network enabled the robot to continue to interact with the human on a given task.

## 6 Discussion

In this paper, we proposed a novel method for linking language to robot behavior, in which the link is encoded as a fixed-dimensional vector in the middle of the RNN forward dynamics that simultaneously represents temporal patterns of interactions as multiple attractors. In the robot experiment, the internal activation representing the link was gained by branching in accordance with the human’s instruction and visual information. From the linking point, the robot immediately generated the appropriate behavior in the subsequent forward calculation, while the internal states move along the second half of the attractor. Moreover, by forming a fixed-point attractor at the initial point, the robot could wait stably in the initial posture for subsequent instruction. Thanks to this structure, which represented not only the link but also some aspects of the interaction, the robot was able to interact with a human on the given task by autonomously switching between recognition, generation, and waiting phases and utilizing the acquired relationship in appropriate contexts. In the following section, we compare the current model with other linking methods and indicate its advantages and disadvantages.

### 6.1 Topologically Organized Linking Structure

The experiment demonstrated that the link was represented as a topologically organized cluster structure on the fixed-dimensional space of the internal states of the top layer. This kind of organization that represents the compositional structure of language could be seen in the study by Sugita and Tani (2005). To link language to robot behavior, they utilized two RNNs that dealt with language and behavior and a module referred to as a parametric bias that bound both RNNs by small-dimensional vectors. As a result of learning that constrained the parametric bias vectors to be equalized for generating corresponding language and behavior, a topological structure representing word meanings and their compositionality was self-organized in the parametric bias space. In their scheme, finding an optimal parametric bias vector (linking point) for translation from a sentence to a corresponding behavior required the iterative back-propagation process. The current experiment showed that a similar kind of topological structure representing the link can be embedded in the forward propagation process by learning.

Representing links as fixed-dimensional vectors in the middle of the dynamics is one of the suitable ways to deal with the “linear nature” of language indicated by Saussure (1959). Language expression is restricted in the sense that a sentence can express a matter only by a linear series of words that extract certain features and that compositionally construct the whole meaning, whereas their order is uncorrelated to the temporal aspect of the matter. In the current case, the “POINT-RIGHT-SLOWLY” behavior cannot be temporally reduced to some parts that correspond to “point,” “right,” and “slowly.” These words express a certain feature relating to whole of the behavior and compose the meaning by being arranged in accordance with a syntactic rule. In other words, the combinatorial structure of language is intrinsically different from of the real world. When one considers dealing with the grounding of language on behavior, both of which have a temporal extent, after accepting the restriction, a method that embeds grounding in a fixed-dimensional space can achieve it in a unified way. In the current experiment, language recognition was embedded as a branching structure that develops a cluster structure corresponding to the behavior to be generated (language recognition and behavior generation). Park and Tani (2015) showed that an MTRNN can recognize human gesture patterns and generate corresponding robot behavior by utilizing a gained fixed-dimensional vector (behavior recognition and behavior generation). Heinrich and Wermter (2014) demonstrated that the MTRNN can generate various sentences from the optimal fixed-dimensional vectors that are gained from a robot proprioception sequence (behavior recognition and language generation). As in these cases, the recognition and generation of undefined-length sequences, including both language and behavior, can be uniformly achieved in RNN dynamics through representations encoding grounding in a fixed-dimensional space.

Such kinds of topological structure can also be seen in the field of NLP. Mikolov et al. (2013) demonstrated that, as a result of training an RNN language model with a corpus, the distributed representations of word meaning were embedded in the high-dimensional space and that some algebraic operations could be executed on the representations (e.g., “King” − “Man” + “Woman” = “Queen”). Although, so far, this kind of analysis has been conducted just in the NLP field, if it is shown in the future that such operations can be similarly applied to linking representations, it will be of great practical use.

### 6.2 Advantages and Disadvantages of the Model

In comparison with other models, the current linking method has both advantages and disadvantages. First, by embedding the link in forward dynamics, the network can translate an instruction into a behavior online with a small calculation cost. Furthermore, another function, autonomously switching between recognition, generation, and waiting phases without explicit cues was achieved by self-organized attractors directly corresponding to the temporal flows of the interaction. However, the model acquired by training with the current data can not translate the behavior sequence to the corresponding sentence. The link is achieved just unidirectionally. Ogata et al. (2007) utilized two RNNs and a parametric bias layer for language–behavior grounding, as in Sugita and Tani (2005). Although their model also required an iterative back-propagation process for translation, the model could perform bidirectional translation, from sentence to behavior and vice versa. In the current scheme, to achieve the translation from behavior to sentence, we have to collect data that consist of behaviors and corresponding sentences in this order, and the network has to make additional paths that correspond to these relations in the dynamical system.

From the opposite point of view, the self-organized dynamics is perfectly dependent on the temporal construction of training sequences. By collecting data constructed as actual temporal flows of the task imposed on the robot, the network seems to acquire an appropriate linking structure without changing the general framework. This leads to the possibility that we can make robots execute collaborative tasks requiring language use, just by giving a certain number of examples as raw sequential data without any preprocessing to construct explicit sets of language and corresponding behavior. In this study, we designed a rather arbitrary task and the training data were collected in an artificial way, such as predesigned trajectories of the joint angles. We need to investigate whether the network can learn from data collected in more natural way, such as direct teaching with real utterances and with raw camera images.

Another considerable point is the stability and the safety of the mechanism. In the current experiment, the robot responded to the human’s instruction even in most of the unexperienced episodes by generalization. It was also confirmed, by adding perturbations, that the initial point was stable to a certain extent. However, we cannot assure that the working of the network is globally stable even for exceptional cases or in a large noise environment because a global analysis of the characteristics of a high-dimensional dynamical system is extremely difficult. The current model continues to work using just the forward calculation; therefore, there is a risk that the dynamics will become unstable in unexpected situations. To ensure safety for practical use, some protective systems that monitor error values or output joint torque should be implemented.

### 6.3 Conclusion and Future Work

In this study, we proposed a novel method for linking language to behavior by means of RNN learning. The robot experiment demonstrated that, as expected, the network self-organized the forward dynamics that directly represented the temporal flows of interaction, and the link was embedded in the middle of the forward dynamics as a fixed-dimensional vector. Thanks to such structure, the robot was able to interact online with a human on a given task by autonomously switching phases and utilizing the acquired relationships in appropriate contexts in the process.

In future work, we plan to conduct other robot experiments to evaluate the following matters. First, the current experiment was limited in a specific simple task, thus we will explore to what extent the task complexity can be scaled up. To scale up the timewise complexity, the implementation of LSTM units, which have recently attracted much interest because of their capacity to process long term dependencies, would be effective. Second, we should evaluate whether the proposed method can be applied to other tasks, such as language generation, behavior recognition, or bidirectional translation. The internal dynamical system of the RNN and the robot’s ability achieved by the proposed method are data-driven. Thus, this approach is compatible with the methodology of deep learning that attempts to make optimal models from large amounts of data. Therefore, the implementation of deep NNs, such as CNNs or auto encoders, would also be effective for the acquisition of dynamical representations of raw sequential data for robots to behave optimally in their placed environment. These deep learning methods also have some drawbacks. For example, it often takes huge amount of time and computer resources, and online or incremental learning could not be performed well unlike the reinforcement learning. In particular, from the point of view of the robot applications, one of the important problems is how to obtain a large amount of training data. We will have to consider using a simulator environment, such as SIGVerse (Tan and Inamura, 2012), for data acquisition.

## Author Contributions

TY, SM, HA, and TO conceived, designed the research, and wrote the paper. TY performed the experiment and analyzed the data.

## Conflict of Interest Statement

The authors declare that the research was conducted in the absence of any commercial or financial relationships that could be construed as a potential conflict of interest.

## Footnotes

^1^Actually, the symbol grounding problem in Harnad’s definition imposes forming categorical representations from the continuous undifferentiated world, on an agent. In this study, we artificially designed the evaluation experiment in a way that the task has categorical structure as described in Section 4, although our model, in fact, can deal with robot behavior in raw motion sequence level. So, we use the term “linking” instead of “grounding,” so that we do not lead the misunderstanding that we have solved the symbol grounding problem in Harnads definition.

^2^In this study, “dynamics” means the time development of the internal states of the RNN context layer that is defined on a fixed-dimensional space. On a discrete dynamical system of forward propagation of an RNN, the current internal states are deterministically calculated from the learnable parameters of the RNN (weights and biases), their own previous states, and external input.

^3^We call adaptive change of characteristics of dynamical system in a certain algorithm “self-organization.” Although the acquired representation in this study is dynamic and thus apparently different from the case of “self-organizing” map (Kohonen, 1982) that forms statically visualized structure, both share the concept that the network autonomously acquires a certain representation of structure or patterns implicitly included in raw data. Tani (2014) also uses self-organization in the same way as us, such as, “self-organized dynamical structure,” “self-organized functional hierarchy.”
